# Chromatin Remodelers: From Function to Dysfunction

**DOI:** 10.3390/genes6020299

**Published:** 2015-06-12

**Authors:** Gernot Längst, Laura Manelyte

**Affiliations:** Biochemistry Center Regensburg, Laboratory of Chromatin Dynamics and Nuclear Architecture, University of Regensbrug, Universitätstraße 31, Regensburg DE-93053, Germany; E-Mails: Gernot.Laengst@vkl.uni-regensburg.de (G.L.); Laura.Manelyte@vkl.uni-regensburg.de (L.M.); Tel.: +49-941-943-2849; Fax: +49-941-943-2474

**Keywords:** chromatin remodeler, cancer, search mechanism, arrest model, non-coding RNA

## Abstract

Chromatin remodelers are key players in the regulation of chromatin accessibility and nucleosome positioning on the eukaryotic DNA, thereby essential for all DNA dependent biological processes. Thus, it is not surprising that upon of deregulation of those molecular machines healthy cells can turn into cancerous cells. Even though the remodeling enzymes are very abundant and a multitude of different enzymes and chromatin remodeling complexes exist in the cell, the particular remodeling complex with its specific nucleosome positioning features must be at the right place at the right time in order to ensure the proper regulation of the DNA dependent processes. To achieve this, chromatin remodeling complexes harbor protein domains that specifically read chromatin targeting signals, such as histone modifications, DNA sequence/structure, non-coding RNAs, histone variants or DNA bound interacting proteins. Recent studies reveal the interaction between non-coding RNAs and chromatin remodeling complexes showing importance of RNA in remodeling enzyme targeting, scaffolding and regulation. In this review, we summarize current understanding of chromatin remodeling enzyme targeting to chromatin and their role in cancer development.

## 1. Introduction

In the eukaryotic nucleus, DNA is packaged into a compact nucleoprotein structure. The basic packaging unit of DNA is the nucleosome core, consisting of 147 bp of DNA tightly wrapped around a histone octamer. Nucleosomal cores are separated by a linker DNA, with a varying length of 20 bp to 90 bp, depending on the organism and cell type [1]. Binding of the DNA to the histone octamer, the bending of the DNA over the protein surface and additional packaging of chromatin into compact higher order structures, thereby masking the regulatory DNA sequences and inhibiting sequence specific recognition by most of the transcription factors. To overcome nucleosomal DNA accessibility problems, cells evolved mechanisms to open higher order structures of chromatin and to (re)move nucleosomes, thereby freeing the DNA element and allowing the binding of sequence specific regulators [2]. In general, two major mechanisms exist which regulate chromatin accessibility: First, histones can be post-translationally modified enabling the recruitment of specific effector proteins to chromatin, thereby changing the activity state of chromatin domains [3]. Second, specific chromatin remodeling complexes displace the histone octamers from DNA or translocate them onto neighboring DNA segments, thereby exposing underlying DNA sequences to sequence specific regulatory factors [4]. The ATP-dependent chromatin remodeling enzymes are highly abundant in the cell, with about one remodeling complex per 10 nucleosomes and also and remarkably diverse [5,6,7].

Chromatin remodeling complexes are multi-protein assemblies containing an ATPase subunit of the Snf2 subfamily that is capable to mobilize the nucleosomes using the energy of ATP hydrolysis and thereby alter the chromatin structure [4]. Additionally, they harbor 2 to 20 non-catalytic subunits that are required for the targeting and regulation of distinct nucleosome positioning activities of remodeling complexes and thereby determine the gene expression program and the cell fate. The many different remodeling enzymes recognize different histone modifications, DNA structures/sequences and RNA signals that target them to specific genomic loci. To recognize these chromatin signals remodeling complexes have dedicated protein domains, termed reader domains. Defined combinations of such a reader domains are required for the specific targeting and regulation of the enzymatic activity. Current data suggest that the accessibility of the DNA through the action of chromatin remodelers is precisely tuned and therefore it is not surprising that the malfunction of chromatin remodeling enzymes trigger cells to undergo cancerogenesis, with this class of enzymes being frequently and specifically mutated in a wide variety of cancers.

## 2. Chromatin Remodelers Fall into Four Families

**Figure 1:**
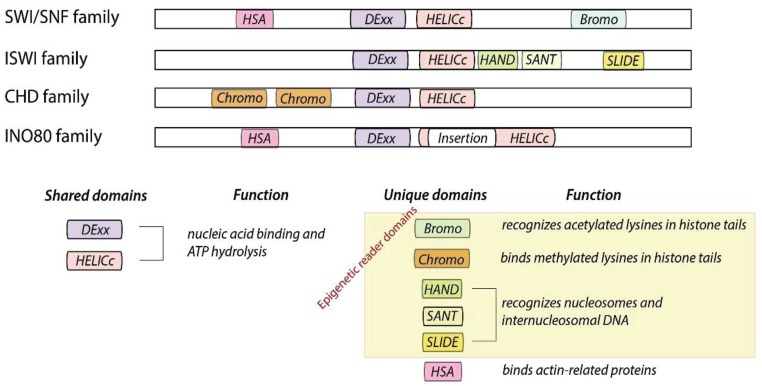
Organization of remodeler families defined by their catalytic and flanking domains. All remodeling enzymes consist of a shared ATPase domain and unique flanking domains.

The catalytic subunit of the remodeling enzymes consists of a conserved ATPase domain with defined flanking domains, and therefore can grouped into four families; e.g., the SWI/SNF, CHD, ISWI and INO80 family (Figure 1). The ATPase domain consists of two tandem RecA-like folds (DExx and HELICc), containing seven conserved helicase-related sequence motifs that classify the enzymes as part of the Superfamily 2 grouping of helicase-like proteins [8,9]. Chromatin remodelers are DNA translocases that use the energy of ATP to create a force to reposition nucleosomes. Unique flanking domains such as bromo- and chromodomain are so called epigenetic reader domains and shown to recognize the post-translational modifications on the histones [10]. The structure of epigenetic reader domains typically exhibits a cavity or surface groove to bind the epigenetic modification. Therefore epigenetic reader domains have emerged as potent targets for therapeutic development [11,12,13].

The **SWI/SNF family** members are defined by the presence of an N-terminally located HSA (helicase-SANT) domain, which is known to recruit actin and actin-related proteins, and a C-terminally located bromo domain, known to bind to the acetylated-lysines of histones [14]. This family of remodeling enzymes was shown to slide and/or to evict nucleosomes from DNA, but lacking chromatin assembly activities. Remodelers belonging to this family are large, multi-subunit complexes containing eight or more proteins. Most eukaryotes utilize two related SWI/SNF type remodelers, built either around the Brm or related Brg1 subunit in humans. Human BAF and PBAF complexes share eight subunits and are distinct by the presence of the subunits BAF180, BAF200 and BRD7 for PBAF and BAF250a for BAF [15]. Variant subunits are thought to contribute to targeting, assembly and regulation of lineage-specific functions of those complexes. For example only PBAF, but not BAF, is capable of facilitating ligand-dependent transcriptional activation by nuclear receptors *in vitro* and to mediate expression of interferon-responsive genes [16,17].

The **ISWI family** (Imitation SWItch) ATPases harbor a C-terminal SANT domain adjacent to a SLIDE domain (SANT-like ISWI), which together form a nucleosome recognition module that binds to DNA and unmodified H4 tails [4]. The ISWI remodeling enzyme in *D. melanogaster*, is known to be present in several chromatin remodeling complexes such as NURF, CHRAC, ACF and RSF. Snf2H and Snf2L are the mammalian homologues of ISWI, which act in the presence of one to three accessory subunits forming different remodeling complexes with different properties. For example, Snf2H is known to interact with Tip5, RSF1 and WSTF proteins to form NoRC, RSF, WICH and ACF complexes. Specialized accessory proteins contain many chromatin binding domains, including histone fold motifs (in ACF/CHRAC), plant homeo domain (in Tip5), bromo domains (in BPTF, Acf1, and Tip5) and additional DNA-binding motifs (HMGI(Y) in NURF301; AT hooks in Tip5). Many ISWI family complexes (ACF, CHRAC, and NoRC) catalyze nucleosome spacing, promote chromatin assembly, compaction of higher order structures of chromatin and are generally involved in transcriptional repression. For example, NoRC action correlates with specific changes in nucleosome positioning at the rDNA promoter region, causing heterochromatin formation and gene silencing [18,19,20]. However, NURF escapes theses general rules by disrupting nucleosome spacing and being involved in ecdysone dependent transcriptional activation, showing that the additional subunits determine enzyme specificity [21]. The steroid hormone ecdysone directly modulates germline stem cells maintenance, activates transcription and proliferation in a cooperation with the NURF remodeler [22].

The **CHD family** (Chromodomain-Helicase-DNA binding) is defined by the presence of two chromo domains, arranged in tandem, at the N-terminal of the ATPase domain. Additional structural motifs are used to further divide the CHD family into the subfamilies CHD1, Mi-2 and CHD7 [9,23]. Members of the CHD1 subfamily contain a C-terminal DNA-binding domain that preferentially binds to AT-rich DNA *in vitro* (members are Chd1 and Chd2 proteins in higher eukaryotes) [24,25]. The crystal structure of the DNA binding domain of Chd1, revealed a SANT-SLIDE like fold. This domain was shown to be required for the remodeling activity of Chd1 *in vitro* and *in vivo* [26]. The Mi-2 subfamily members contain a pair of PHD domains (plant homeodomain) in their N-terminal part (human Chd3 and Chd4, also known as Mi-2α and Mi-2β, respectively), implicated in nucleosome binding [27]. The CHD7 subfamily members have additional C-terminal domains, like the SANT or BRK domains (Chd5 to Chd9 proteins). The biological properties of CHD family members are highly heterogeneous. Some exist as monomers *in vivo*; others are subunits of multiprotein complexes, many of which have not yet been fully characterized [28]. The best studied is the NURD (nucleosome remodeling and deacetalase) complex, containing Chd3/Chd4, histone deacetylases (HDAC1/2) and methyl CpG-binding domain (MBD) proteins. It was shown to be involved in transcriptional repression of a specific set of genes during *C. elegans*, *D. melanogaster* and mammalian development [28]. Chd1 together with Isw1 are also termed nucleosome-spacing enzymes that are required to maintain nucleosomal organization in yeast [29].

The specific feature of the remodeling enzymes belonging to the **INO80 family** (inositol requiring 80) is the split ATPase domain. This unique module retains ATPase activity, and acts as a scaffold for the association with the RuvB-like proteins, Rvb1 and Rvb2. RuvB is a bacterial ATP-dependent helicase that forms a double hexamer around Holliday junctions to promote their migration during homologous recombination [30]. Unlike remodelers of other families, the INO80 complex exhibits DNA helicase activity and binds to specialized DNA structures *in vitro.* These DNA structures resemble Holliday junctions and replication forks consistent with the function of the complex in homologous recombination and DNA replication [31,32]. Yeast INO80 was shown to control the genome-wide distribution and dynamics of the histone variant H2A.Z. INO80 and Swr1 were shown to exhibit histone-exchange activity, being capable to replace nucleosomal H2A.Z/H2B with free H2A/H2B dimers [33,34]. Both remodeling complexes can slide nucleosomes *in vitro* on a reconstituted chromatin template and evict histones from DNA [35,36,37]. In addition to the role of INO80 in recombination and DNA replication, it is suggested to regulate about 20% of the yeast genes and to participate in DNA double-strand break repair via the interaction with γ-H2AX and recruit the MRX and Mec1 complexes to the DNA damage site [33].

## 3. Translocation Mechanism

Chromatin remodelers use the energy of ATP hydrolysis reposition nucleosomes on the DNA without dissociating from the histone octamer [38,39]. All proposed models for nucleosome sliding by chromatin remodelers assume that only a minor fraction of the 358 direct and indirect histone-DNA interactions are disrupted at a given time of the reaction, since the energy of ATP hydrolysis would not be sufficient to fully disrupt the nucleoprotein structure [40,41]. One of the first mechanisms proposed, was the “twist diffusion model” suggesting the rotation of DNA in 1 bp intervals over the histone octamer surface. Thus, a single base pair distortion is continuously propagated through the nucleosome, transiently storing one additional basepair in the realm of the nucleoprotein structure. This model is supported by the nucleosomal crystal structures exhibiting such a single-basepair “twist defect” [40,42]. However, several studies could not confirm such a translocation model. Experiments using nicked or gapped DNA substrates that uncouple DNA rotation mediated processes still allowed SWI/SNF and ISWI dependent nucleosome remodeling, arguing against a sole twist-diffusion mechanism [43,44,45].

Alternatively, it was suggested that nucleosomes are repositioned according to the “loop recapture model”, proposing a detachment of a DNA segment from the histone octamer surface at the entry site of the nucleosome. The exposed octamer surface would interact with more distant regions of the DNA molecule, resulting in the formation of a DNA loop on the histone octamer surface. This DNA loop would translocate over the octamer surface in an energy-neutral process, by releasing and rebinding adjacent sequences on the protein surface. DNA loop propagation would change the translational position of the nucleosome, according to the size of the DNA loop [46]. This model is strengthened by biochemical and single molecule studies. ACF remodeling complex was shown to cause the unwrapping of DNA, roughly 20 and 40 bp, from the nucleosomal border [47]. ATP dependent translocation of SWI/SNF and RSC on DNA and nucleosomal templates produces DNA loops and nucleosome remodeling by RSC was shown to produce a remodeled intermediate containing internal DNA loops [48].

Nucleosomal translocation and its corresponding step-size correlates with the size of the DNA loop, a parameter that depends on the nature of the remodeling enzyme. Single molecule studies with the remodeling complex ACF suggested an initial step size of 7 bp and subsequent steps of 3–4 bp [49], whereas RSC was shown to exhibit a step size of 2 bp [50]. Within a strong nucleosomal positioning sequence both, recombinant *Drosophila* Mi-2 and native RSC from yeast, repositioned the nucleosome by 10 bp intervals, which are intrinsic to the positioning sequence. Furthermore, RSC-catalyzed nucleosome translocation was noticeably more efficient when beyond the influence of this sequence. Interestingly, under limiting ATP conditions RSC preferred to re-position the nucleosome by 20 bp steps within the positioning sequence, suggesting that native RSC preferentially translocates nucleosomes in 15 to 25 bp long DNA steps [51]. Lately, it was proposed that loops do not freely diffuse over the histone octamer surface but rather feed through specific restriction points by threading past fixed constrictions [48].

## 4. Targeting Chromatin Remodeling Enzymes to Specific Genome Locations

The human genome is packaged into some 30 millions of nucleosomes that have to be organized into functional chromatin domains with specific local structures. Binding to identify target sites or detecting nucleosomes that have to be repositioned is achieved by plenty of different targeting signals [52].

### 4.1. Mechanisms

Many proteins in the nucleus, including many remodeling enzymes are highly mobile as revealed by fluorescence recovery after photobleaching (FRAP) experiments. For proteins that do not interact with any cellular structures, FRAP kinetics are a direct reflection of their translational motion properties. In contrast, proteins that bind to immobile structures such as chromatin, exhibit a slower overall mobility. The mobility of ISWI family remodelers Snf2H, Snf2L and Snf2L + 13 (an ATPase inactive variant of the Snf2L) was studied in living U2OS cells. During G1/2 phase only 1%–4% of the enzymes were immobilized [52], whereas the rest could be fitted by the free-diffusion model, suggesting only transient binding events. ChIP-Seq analysis of remodeling enzymes supports the transient binding events, where the localization pattern of wild-type Isw2p did not correlate with known sites of Isw2 function *in vivo*. However, the catalytically inactive Isw2p–K215R was preferentially enriched at the known Isw2 target sites. This suggests, that in the absence of ATP hydrolysis the target sites remain high affinity binding sites, whereas the ATPase active enzyme does not bind any longer to the remodeled nucleosomes, *i.e.*, Converting them into low affinity binding sites [6,52,53]. These results indicate a continuous sampling mechanism, by which the remodeler continuously screens the genomic nucleosomes for “good” substrates (high affinity binding sites), converting them into the “bad” ones (low affinity binding sites). The term “good” substrates means that this specific nucleosome is bound with high affinity by the remodeling enzyme, changing its position until it is placed at site where it exhibits low affinity binding towards the remodeling enzyme (“bad” substrate). The particular remodeling complex has its own flavor of “good” and “bad” substrates. It seems that most of the binding events are unproductive, meaning that the remodeling reaction does not occur. According to the high remodeling enzyme concentrations in the cell (in the range of μM) and the short chromatin bound residence times (around 100 ms), an average sampling time to screen the positions of all 30 million nucleosome positions is in the range of tens of seconds to minutes was calculated for Snf2H containing remodeling complexes. Thus, a combination of high remodeler concentrations, short residence times of the chromatin bound states and common diffusion in the intervening periods, appears to be an efficient mechanism to keep nucleosome positions in proper places [52,54].

A kinetic proofreading mechanism can be used to describe the action of remodelers, where “good” substrates are characterized by a high affinity of the remodeler for the nucleosome substrate (low value of Michaelis-Menten constant *K*_M_) and a high catalytic conversion rate *k*_cat_, efficiently moving the nucleosome to the end position of the translocation reaction. Thus, the *k*_cat_/*K*_M_ ratio is high, as expected, for an efficient catalytic process. The opposite would be true for “bad” nucleosomal substrates, *i.e.*, having a low *k*_cat_/*K*_M_ ratio. According to this model, remodeler bind to “good” substrates and move them as long, as they are converted to “bad” substrates, exhibiting a lower affinity for the remodeler. The remodelers are released from the low affinity substrates, a mechanism termed “release model”. In an alternative “arrest model”, all nucleosomal substrates are recognized with the similar affinity, but remodeler has a slow translocation rate on a “bad” substrate. *In vitro* binding assays showed that the Chd1, ACF and NoRC complexes were bound with lower affinity to the nucleosomes at positions that reflected the end points of the remodeling reaction, suggesting that those enzymes function according to the release model [6,55].

In parallel with the continuous sampling mechanism, remodeling complexes are engaged by specific recruitment or immobilization at specific target sites. The respective mechanisms are described in Chapter 4. For example, when cells were treated with dexamethasone, Brg1 and Brm were concentrated in a single spot in the nucleus, as revealed by immunofluorescence. The site coincided with the multimerized MMTV DNA and RNA FISH signals, showing that the enzymes are recruited to the MMTV array in a hormone-dependent manner. In this case the recruitment of the SWI/SNF machine results in the maintenance of an active chromatin structure that is compatible with transcription [56]. In other cases, like the nucleolar remodeling complex NoRC recruitment to the rRNA genes, continuous targeting results in gene repression via changes of the promoter nucleosome positioning that are in-compatible with transcription initiation factor binding and further leads to the heterochromatin formation [57,58].

Cells express a plethora of different remodeling complexes that act simultaneously on the cellular chromatin. The remodeler complexes diffuse freely through the nucleus, searching for “good” nucleosomes. “Good” nucleosomal substrates for the one machine may represent “bad” substrates for the other machine, suggesting that an active, free diffusing pool of different remodeling complexes with distinct activities would continuously change the local chromatin structure. Upon specific signals individual machines are recruited to the specific sites to establish local chromatin structures correlating with a persistent activation or repression of certain DNA dependent processes. We hypothesize that the mixture and the individual concentrations of remodeling complexes in the cell, would establish global chromatin architecture that could respond to the one or other signal and being not responsive for other signaling pathways. Overall the action of the diverse remodeling complexes suggests that chromatin is bar-coded by site-specific nucleosome positions and potentially these combinations define the identity of a cell type.

### 4.2. Targeting Signals Recognized by Chromatin Remodelers

This chapter summarizes the mechanisms and modules that target remodeling enzymes to chromatin. Currently post translational modifications of histones, histone variants, DNA sequence/structure, RNA molecules and transcription factors are known to recruit remodeling enzymes to specific target sites in the genome (Table 1).

**Table 1:** Targeting signals recognized by chromatin remodelers.

Molecular Interaction	Description Selected Examples	Refs.
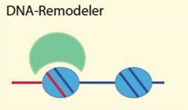	DNA secondary structure, G-quadruplexes, DNA linker length, DNA modification	[6,51,55,59]
	Examples: Brg1, Chd1, ISWI, ACF, NURF, Mi-2, Snf2H, Tip5/NoRC, ATRX, NURD, RSC	
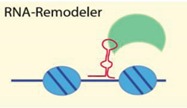	Noncoding RNA secondary structure	[55,61,62,63,64]
	Examples: ISWI, Tip5/NoRC, Brg1	
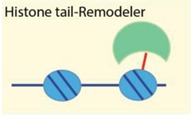	H3 acetylation recognized by bromo domains, methylation—Chromo domains, PHD. H4 tails are required for SANT domain binding.	[65,66,67,68,69,70,71]
	Examples: Tip5/NoRC, RSC, ISWI, Chd4	
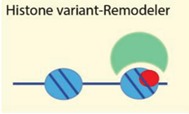	Histone variants mH2A, H2A.Z, H2A.X	[72,73,74,75]
	Examples: ATRX, Ino80, WICH, Lsh	
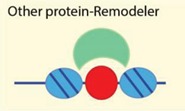	Transcription factors and other protein-protein interactions	[28,76,77,78]
	Examples: NoRC, NuRD, Snf2H	

Examples: NoRC, NuRD, Snf2H

#### 4.2.1. DNA Sequence, Structure and Modification

**Figure 2:**
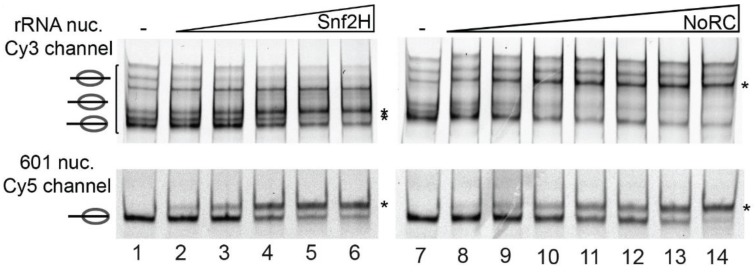
DNA-sequence and the subunit composition of the chromatin remodeling complexes determine the affinity and outcome of the reaction. The 280 bp ribosomal RNA gene promoter sequence and the 208 bp 601 sequence were reconstituted into the nucleosomes using the salt dialysis method. The rRNA gene promoter sequence exhibits several translational positions of the nucleosome, whereas the 601 exhibits a single nucleosome position, as described (lane 1). The differentially labeled nucleosomal DNA templates were mixed and either incubated with increasing concentrations of Snf2H or NoRC in the presence of ATP (lanes 2–6 and 7–14) [55]. Nucleosome positions were analyzed by native polyacrylamide gel electrophoresis.

Mechanistical analysis of the nucleosome remodeling process revealed that binding of a remodeling complex to a mononucleosomal substrate results in a specific and ATP-dependent repositioning of the nucleosome on the DNA [38,79]. Remodeling complexes bind nucleosomal templates reconstituted on different DNA sequences, or nucleosomes exhibiting distinct positions on the same DNA sequence with different affinity and remodeling efficiency. For example, NoRC binds with higher affinity to its cellular target site and therefore the nucleosome remodeling reaction was more efficient on the rRNA gene promoter sequence than on the 601 nucleosome positioning sequence (Figure 2). Moreover, the final position of the nucleosome after remodeling with Snf2H or NoRC (Snf2H + Tip5) were different, suggesting that the accessory subunit determines targeting and remodeling outcome [6,55]. Genome-wide studies compared four different remodeling complexes, NURD, (P)BAP, INO80 and ISWI, and, similarly, it was observed that each remodeler exhibits a unique set of genomic targets correlating with distinct chromatin signatures [80].

A comprehensive study comparing seven different remodeling activities, ACF, ISWI, Snf2H, Chd1, Mi-2, Brg1 and NURF, revealed different remodeling outcomes for all of these enzymes, suggesting differential binding affinities of these enzymes to the individual nucleosome positions [6]. Thus, these data suggest that the remodelers are capable to recognize the underlying DNA sequence/structure and accordingly establish specific chromatin structures.

The remodeling complexes contain DNA-binding motifs that are present in the catalytic or/and in accessory subunits. For example, catalytic subunit Snf2H contains a SANT-SLIDE domain and in addition the WAC and AT hook motifs are present in the Acf1 and Tip5 subunits of the ACF [4,76,81,82,83,84]. These modules allow the recognition of DNA sequences and determine the outcome of a remodeling reaction [51,85,86,87]. For example, Chd1, with deleted DNA binding domains moved the nucleosome to the DNA ends with reduced kinetics. Interestingly, the incorporation of *E.coli* transcription regulator AraC DNA-binding domains to the C-terminus of Chd1, improved nucleosome sliding and moved the nucleosome to the center [88]. Thus, it seems that the DNA binding capability of the remodeler directly influences the directionality of nucleosome sliding. Additionally, the maintenance of internucleosomal distances depend on the DNA binding domains of the enzymes. ACF interacts with linker DNA and is capable to sense their length [89]. This structural element appears to play a key role in the positioning of nucleosomes in regular arrays, as the remodeler dependent nucleosome movement is oriented towards the longer flanking DNA [90]. Similarly, the Chd1 and NoRC remodelers were described to sense the length of linker DNA [55,87]. The DNA sequence length variations as small as 5 bp can also control the destination of the nucleosomes as it was shown for RSC and Mi-2 remodelers [51].

Moreover, unusual DNA structures like quadruplex forming DNA elements could represent specific targeting signals. ATRX is involved in deposition oh H3.3 histone variant in telomeres that harbor G-rich repeat sequences, which are prevalent in the telomeric sequences. These repeat sequences are likely to form G-quadruplex (G4) structures, and ATRX preferentially binds to such a G4 structures *in vitro*. Such alternative DNA structures are believed to destabilize the genome and it is enticing to think that ATRX is responsible for stabilizing G-rich regions of the genome by remodeling G4 DNA and incorporating H3.3-containing nucleosomes [59,91].

Mbd3 (methyl-CpG-binding domain protein 3) localization requires Tet1, suggesting that hydroxymethylation plays a role in Mbd3 recruitment *in vivo*. It was shown that Mbd3 binds preferentially to 5hmC-modified DNA, when compared to 5mC modified DNA, suggesting that Tet1 mediated hydroxymethylation serves to recruit the Mbd3/NURD complex. Thus Mbd3/NURD could be an effector that mediates gene expression upon DNA-hydroxymethylation [60].

The TAM domain (MBD-like) in Tip5 is related to Mbd3. However, the noncatalytic subunit of the NoRC complex, does not recognize methylated DNA, but binds to RNA and more specifically to the pRNA (promoter RNA), which is complementary to the rRNA gene promoter sequence. The pRNA is folded into the hairpin-like structure and is apparently required to recruit NoRC to the rRNA gene promoter, where it serves as a scaffold for other proteins [55,61,62,76,92].

#### 4.2.2. RNA

**Figure 3:**
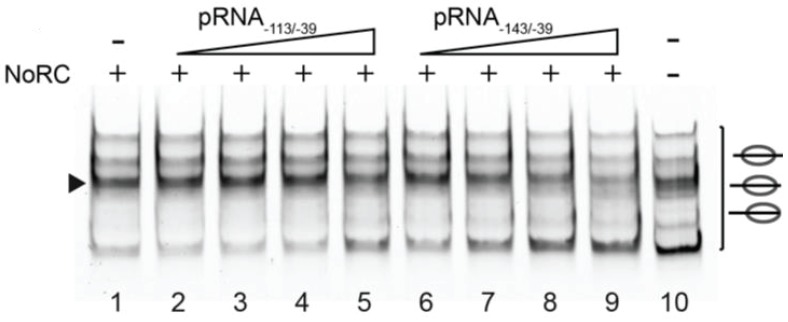
pRNA inhibits the activity of NoRC. Nucleosomes assembled on the −190 to +90 rDNA DNA fragment were incubated with NoRC, ATP and increasing concentrations of pRNA_−143/−39_ and pRNA_−113/−39_. The remodeling reactions were analyzed by EMSA. The arrowhead indicates the nucleosome at the −120/+27 position [55].

Non-coding RNAs play a significant role in the epigenetic control of gene expression and chromatin dynamic. Lots of identified non-coding RNAs have unknown functions, however there is mounting data for their role in gene silencing through the interaction with chromatin remodelers. First identified was promoter RNA (pRNA) originating from intergenic spacer that separates rRNA genes and through interaction with Tip5 (non-catalytic subunit of NoRC complex) recruits NoRC complex to the rRNA gene promoter and leads to silencing [61,62]. In this case, the pRNA most probably has a targeting function, since it competes with the nucleosomes for NoRC binding and in the presence of pRNA NoRC exhibit no nucleosome repositioning activity (Figure 3) [55].

The ATPase activity of *Drosophila* ISWI is regulated by the hsrω-n ncRNA, essential for the assembly and organization of the hnRNP-containing omega speckles. *In vivo* data suggest that omega speckle nuclear organization depends on ISWI function, since in the absence of ISWI or hsrω-n ncRNA omega trail-like structures are not formed [63]. Furthermore, the interaction between chromatin remodelers and non-coding RNAs can be implicated in cancer and other diseases. Urothelial carcinoma associated 1 (UCA1) long non-coding RNA, identified in bladder cancer tissues, interacts with Brg1 and, similarly as observed with NoRC at the rRNA promoter genes, was shown to inhibit the binding of remodeler to the p21 promoter [64]. Another study shows that SChLAP1, a long noncoding RNA frequently expressed in aggressive prostate tumors, drives cancer by directly disrupting Snf5, a core subunit of the SWI/SNF complex [93,94]. Another long-noncoding RNA, Mhrt, required for proper heart function, was shown to prevent Brg1 from recognizing its genomic.

DNA targets. Inhibiting chromatin targeting and gene regulation by binding to the helicase domain of Brg1, a domain that is crucial for tethering Brg1 to chromatin [95].

#### 4.2.3. Histone Modifications

Chromatin remodeling complexes contain epigenetic reader domains that recognize covalent modifications on histone tails, allowing the targeting to specifically modified chromatin domains and thereby enabling the establishment of a remodeler dependent nucleosomal positioning landscape. Acetylated lysines on the histone tails are recognized via bromo domains [96]. Binding of the bromo domain of Tip5, the large subunit of NoRC, to H4K16ac is a prerequisite for NoRC function. A point mutation within the bromodomain impairs the association of NoRC with chromatin, prevents heterochromatin formation, and abolishes transcriptional repression [66]. Recently, *in vitro* experiments revealed that the bromo domain of Tip5 interacts also with H3K14ac [67]. Nucleosomes acetylated on H3K14 position have a higher affinity for the chromatin remodeling complex RSC and show increased DNA repair efficiency on chromatin [97]. H2A.Z deposition is controlled by SWR-C chromatin remodeling enzymes that catalyze the nucleosomal exchange of canonical H2A with H2A.Z. It was reported that acetylation of histone H3 K56Ac alters the substrate specificity of SWR-C, leading to promiscuous dimer exchange in which either H2A.Z or H2A can be exchanged from nucleosomes [98]. The tandem PHD domain of DPF3b presents an alternative of recognition of acetylated lysines on the histone tails, that functions in association with human BAF chromatin remodeling complex and initiate gene transcription during heart and muscle development. DPF3b was reported to bind histones H3 and H4 in an acetylation-sensitive manner [99,100].

Methylated lysines on the histones are recognized by chromo domains and plant homeodomains (PHD). Human Chd1 protein interacts with H3K4me2/3 via its double chromo domains, which fold into a functional unit. On the other hand, nucleosomal H3K4 methylation reduces the affinity of the NuRD complex for H3 tail binding. It was shown that the second PHD finger of Chd4 preferentially interacts with unmodified H3K4 and H3K9me3 tails [69,70,71]. Rice Chd3 protein CHR729, required for many aspects of plant development, can interact with dimethylated histone H3 lysine 4 (H3K4me2, a mark associated with moderately expressed or repressed genes) and with trimethylated histone H3 lysine 27 (H3K27me3, a mark associated with repressed genes), respectively, through the chromodomains and the plant homeodomain (PHD) finger of the protein [101]. However, it seems that the PHD domain of Tip5 reconizes monomethylated and unmodified H3K4 tail [67]. By contrast, the SANT domain of the ISWI type enzymes is known to interact with non-modified histone tails. The versatility of reader domains in the recognition of histone tails and their modifications plus their different combinations and specificities in chromatin remodelers suggest a complex pattern of remodeling enzymes targeting and marking of local chromatin structure.

#### 4.2.4. Histone Variants

Non-canonical histone variants differ from the canonical histones at the level of their primary sequence, which can range from a few amino acid changes to large domains. Chromatin remodelers act as assembly or exchange factors that determine the eviction of nucleosomes from specific regions in the genome, create open DNA regions that are targeted by histone chaperones for the specific type nucleosome incorporation [102,103]. Yeast INO80 controls genome-wide distribution of H2A.Z and thereby facilitates DNA repair, transcription and replication. It was demonstrated that INO80 has a histone-exchange activity in which the enzyme can replace nucleosomal H2A.Z/H2B with free H2A/H2B dimers. Genetic interactions between ino80 and htz1 support a model in which INO80 catalyzes the removal of unacetylated H2A.Z from chromatin as a mechanism to promote genome stability [34]. ATRX was identified as a novel binding partner for the histone variant mH2A and was found to negatively regulate mH2A incorporation and the transcription of *HBA* genes [73]. ATRX-dependent deposition of H3.3 into heterochromatin is generally required to maintain the memory of silencing at imprinted loci throughout the genome [104]. Transcription factor Foxa2 and H2A.Z recruit nucleosome disassembly complexes NAP111/SWI/SNF/INO80 and regulate nucleosome depletion and gene activation during ES cell differentiation [105]. Chromatin remodeling enzymes are also involved in the modification and dynamics of the histone variant H2A.X, which is phosphorylated upon DNA damage and repair. The INO80 complex is recruited to a HO endonuclease-induced DSB through interaction with phosphorylated histone H2A (gamma-H2AX), induced by the DNA damage. This interaction requires Nhp10, an HMG-like subunit of the INO80 complex [72]. The WICH (WSTF-Snf2H) chromatin-remodeling complex exhibits a novel kinase domain capable to phosphorylate Y142 on H2A.X. Both proteins, WSTF and Snf2H were also shown to bind to H2A.X in co-immunoprecipitation experiments [74]. In addition, it was recently shown that the activity of the Lsh remodeling enzyme is necessary for the efficient phosphorylation of H2A.X at DNA double-strand breaks and the successful repair of DNA damage [75].

#### 4.2.5. Targeting to Chromatin by Sequence Specific Binding Proteins

The DNA-sequence dependent recruitment of remodelers is not necessarily mediated by the remodeling complex subunits themselves but can also occur via transient interactions with other sequence specific DNA binding proteins. Fore example, Brg1 and Brm, core components of the mammalian chromatin remodeling complex and histone H3K4 methylation complex (Ash2, absent, small, or homeotic discs 2, or Ash2 and WD domain repeat 5, or Wdr5) were recruited to the endothelin promoter region in endothelial cells in response to Angiotensin II stimulation. Angiotensin II induces cardiac hypertrophy and fibrosis in part by stimulating endothelin transcription [106]. The NuRD complex physically interacts with FOG-2, a multi-zinc finger protein that binds the transcriptional activator GATA4 and modulates GATA4-mediated regulation of target genes during heart development. FOG-2/NuRD interaction is required for repression of GATA4 activity, cardiomyocyte proliferation by directly down-regulating the cell cycle inhibitor Cdkn1a during heart development [107]. SIRT6 recruits the chromatin remodeler SNF2H to DSBs and focally deacetylates histone H3K56. Lack of SIRT6 and SNF2H impairs chromatin remodeling, increasing sensitivity to genotoxic damage and recruitment of downstream factors such as 53BP1 and BRCA1 [77]. NuMA coimmunoprecipitates with Snf2H, regulates its diffusion in the nucleoplasm and controls its accumulation at DNA breaks. Consistent with NuMA enabling Snf2H function, cells with silenced NuMA exhibit reduced chromatin decompaction after DNA cleavage, lesser focal recruitment of homologous recombination repair factors, impaired DNA double-strand break repair in chromosomal (but not in episomal) contexts and increased sensitivity to DNA cross-linking agents [78].

## 5. Implication in Cancer

**Figure 4:**
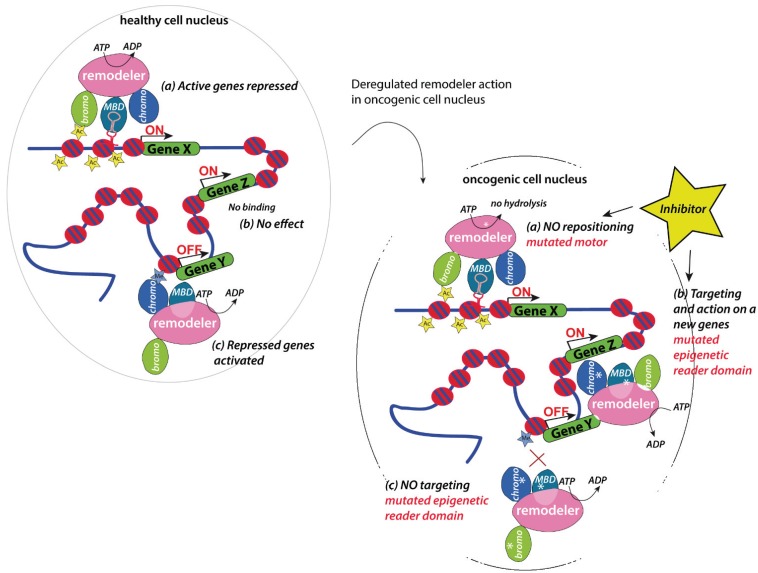
Model describing the potential activities of deregulated chromatin remodelers in an oncogenic cell. Chromatin remodelers that involved in the repression of active genes, or maintaining genes repressed (**a**), might loose their activity on the particular gene. The same could occur with activities acting on repressed genes (**b**). Mutations in the reader domains could result in targeting of the complexes to non-genuine targets altering its activity status (**c**). To prevent the recruitment of inactive remodelers or to inhibit the mis-targeting of the enzymes, the currently available bromo domain inhibitors (marked with a star) may be useful.

Identity and function of cells is affected by the gene expression program, which is modulated by chromatin accessibility, nucleosome positioning and histone modifications. Chromatin remodelers function as gatekeepers and constitute the major determinant of accessibility of DNA-binding factors and to ensure the variety of biological functions of the cell [108]. It is hypothesized that alterations of the chromatin organization and gene expression programs are driven by mutations and aberrant expression of chromatin remodeling factors, which may present crucial triggers of tumorigenesis in at least some tumor types [109]. Thus, the perturbation of chromatin remodeling complexes is an emerging theme in malignant transformation and progression (Figure 4).

Cancer genome sequencing projects revealed that members of the SWI/SNF families are predicted to have driver function in various cancers [109] and 20% of all human tumors contain mutations in at least one member of the SWI/SNF complex. In comparison, the known tumor suppressor gene p53 was mutated up to 26% in various cancers. The cancers with the highest SWI/SNF mutation rates were ovarian clear cell carcinoma (75%), clear cell renal cell carcinoma (57%), hepatocellular carcinoma (40%), gastric cancer (36%), melanoma (34%), and pancreatic cancer (26%) [110,111]. At least nine subunits of SWI/SNF complex have mutation frequencies significantly higher than background, suggesting that these genes present “driver” rather than “passenger” of tumor progression. For example, Brg1 is one of the most commonly mutated subunits across cancer, occurring at a frequency of about 3% in all cancers and arising regularly in non-small cell lung carcinoma (NSCLC), Burkitts lymphoma and medulloblastoma, while also occurring in melanoma, pancreatic adenocarcinoma, ovarian clear cell carcinoma and other tumor types [110,112]. Also 69% of SCCOHT (small cell carcinoma of the ovary of hypercalcemic type) carry germline and somatic mutations of Brg1 in addition to complete loss of the protein in 82% of the cases [113,114]. Loss-of-function SWI/SNF subunit mutations seem most prevalent in cancer, *i.e.*, SCCOHT, NSCLC, point mutations have also been described, such as a small number of SMARCA4 missense mutations in medulloblastoma [115]. It is not yet understood whether these point mutations also result in loss of function of the protein, as in a classical tumor suppressor, or whether they result in partial loss, or even potential oncogenic gain-of-function effects. Looking forward, elucidating the effects of these point mutations will likely provide further mechanistic understanding of the cancer-promoting activity of SWI/SNF mutations [116]. Interestingly, for the proliferation and viability of leukemia cells the presence of the Brg1 subunit is often critical, a function distinct from its tumor suppressor role described previously in other cancers. This observation was related at least in part to a unique role of Brg1 in the maintenance of Myc expression in leukemia cells [117]. Functional studies have so far identified dual roles for Brg1 in both differentiation and cell adhesion/migration. In embryonic stem cells (ESCs), inactivation of Brg1 leads to defective self-renewal and promotes differentiation, while overexpression enhances the epigenetic reprogramming of fibroblasts into induced pluripotent stem (iPS) cells, possibly through increased OCT4 binding to target genes [118]. Still, the mechanisms by which Brg1 mutations contribute to tumorigenesis are largely unknown [15,110,119,120]. Brg1 possesses tumor suppressor functions, whereas BRM loss is a contributing factor and potential marker of tumorigenesis in lung, prostate and gastric cancers [121]. Inactivating mutations of ARID1A are prevalent in a wide variety of cancers *i.e.*, 45% of ovarian clear cell and endometrioid carcinomas [122,123], 19% gastric cancers [124], 19% bladder cancers [125], 14% hepatocellular cancer [126]. Moreover, ARID1A appeared as a useful marker of malignancy in peritoneal washings for endometrial carcinoma [127]. Loss of ARID1A expression is associated with poor prognosis in patients with stage I/II clear cell carcinoma of the ovary [128], small intestinal carcinoma [129], and clear cell renal cell carcinoma [130].

NuRD has been shown to have opposing effects in cancer, both promoting and inhibiting tumor growth and metastasis depending on different tissue. To some extent, these paradoxical effects might be explained by the ability of NuRD to associate with or modulate the activity of both tumor suppressors (eg, p53) and oncogenic factors (eg, Bcl-6) [131]. MTA1 expression is elevated in breast and other tumors, and correlates with an increased risk of metastasis and poor outcome [132]. MTA1 is thought to be a downstream effector of the Myc oncogene, which could explain why increased levels of MTA1 are associated with high tumor grade and invasiveness in a variety of cancers [133]. Whole exome sequencing of serous uterine tumors, a highly aggressive form of endometrial cancer, identified Chd4 as one of the proteins containing high frequencies of somatic mutations [134]. Chd4 was highly mutated in serous tumors (17%) and was also mutated in clear-cell (4%), endometrioid (7%) and mixed-histology (11%) tumors. It was found that 80% of Chd4 missense mutations, including those affecting an Arg1162 hotspot, were predicted to have an impact on protein function. Half of all Chd4 mutations affected the ATPase/helicase and HELICc domains. These observations lead to the speculation that somatic mutations affecting the ATPase/helicase domain of Chd4 present driver mutations in endometrial cancer [137]. Moreover, the cancer sequencing data deposited by the ICGC consortium reveals that Chd4 is mutated in other cancers such as thyroid (27%), ovarian (12%), malignant lymphoma (11%), gastric (10%), skin (10%), bladder (10%) and numerous, cancer-associated, single somatic mutations were identified. It was found that depletion of CHD4 is synergistic with DNMT inhibition in reducing the viability of colon cancer cells in correlation with reactivation of tumor suppressor, suggesting that their combined inhibition may be beneficial for the treatment of colon cancer. Since Chd4 has ATPase activity, the observed data identify Chd4 as a potentially novel drug target in cancer [135]. Chd3 is associated with Hodgkin’s lymphoma and Chd5 is associated with neuroblastoma, a malignant neoplasm of the peripheral sympathetic nervous system frequently affecting infants and children [136].

The abundance of Baz2A was found to be increased and proposed to be biomarker for pancreatic cancer [137]. Baz2A (non-catalytic subunit of NoRC complex) is a key epigenetic regulator linking aberrant DNA methylation and outcome in prostate cancer [138]. The remodeling complex NoRC was shown to be involved in Ras dependent tumors [139] and overexpression of miRNAs, regulating the expression of BAZ2A, result in progression to metastasis in prostate cancer and may also play a role in chronic lymphocytic leukemia [140].

### 5.1. Chromatin Remodelers as Regulators of Master Regulators

SWI/SNF, consistent with its role as a potent tumor suppressor, is a key regulator of cellular proliferation and that its loss stimulates activation of proliferation-associated genes. Further, at least in the case of Brg1, its loss may lead to impaired sensing of intercellular signaling and disrupted control of migration. The Gene ontology analyses demonstrate that, despite the fact that MEFs undergo cell-cycle arrest following inactivation of either Snf5 or Brg1, loss of these subunits promotes cell-cycle progression. Thus, the aberrant proliferative drive caused by SWI/ SNF mutation may trigger arrest at a cell-cycle checkpoint [141]. Consistent with this, it was found that inactivation of p53 dramatically accelerates the onset of cancers caused by Snf5 inactivation [142,143].

### 5.2. Therapeutic Opportunities

Targeting subunits and domains of chromatin remodelers is currently being evaluated as a major therapeutic strategy in the prevention and treatment of human cancers. Chromatin remodelers harbor epigenetic reader domains that arise as novel drug targets. JQ1 molecule, inhibiting BRD4 protein through its bromo domain, was the first discovered inhibitor, which is already in clinical trials [144]. Both JQ1 and the novel BET inhibitor I-BET151 showed remarkable efficacy *in vitro* and *in vivo* against MLL fusion leukemia, resulting in the rapid induction of cell-cycle arrest and apoptosis. Both studies highlighted a reduction in expression of critical regulators of transformation, including MYC BCL2, and CDK6 (a cyclin-dependent protein kinase), after treatment with a BET inhibitor [145]. A similar approach hold true for chromatin remodelers. The structural genomic consortium (SGC) crystalized a variety of bromo domains [146] and screened for the specific inhibitors. GSK2801, a potent, selective and cell active acetyl-lysine competitive inhibitor of Tip5 (BAZ2A) and BAZ2B bromodomains was recently developed. A pharmacokinetic study in mice showed that GSK2801 had reasonable *in vivo* exposure after oral dosing, with modest clearance and reasonable plasma stability. Thus, GSK2801 presents a versatile compound for cellular and *in vivo* studies, first to understand the role of BAZ2 bromodomains in chromatin biology [147]. Brm and Brg1 bromodomains are also potentially drugable with specific Pfi-3 inhibitor (http://www.thesgc.org). However, it still remains a challenge to develop specific inhibitors for the homologous domains such as Brm and Brg1, Baz2A and Baz2B. On the other hand, it was shown that Brm and Brg1 display differential transcription factor interactions, in part due to structural differences, but it remains to be determined whether such structural differences can be effectively exploited for inhibitor targeting [116].

## 6. Conclusions

Global chromatin structure is a result of the combination of chromatin remodelers present in the cell. The ability to form various complexes with different activities and the concentration of the remodelers influences the nucleosomal positions genome-wide. Much data have been accumulated from *in vitro* studies addressing the mechanism of these enzymes, but recent studies have begun to reveal how these enzymes find their place of action in order to modify chromatin structure in cells. From our current knowledge, it seems that the local chromatin structures undergo continuous proofreading to fix cell type and specific chromatin structures. On the other hand, perturbation of this balanced regulation of chromatin accessibility results in sustained chromatin and gene expression changes that can lead to oncogenic transformation. Cancer sequencing projects identify chromatin remodelers as drivers of tumorigenesis, placing them on top of the list as potential drug targets in cancer treatment.
